# Supplementary material for: SARS-ANI: a global open access dataset of reported SARS-CoV-2 events in animals
Source: Sci Data. 2022 Jul 23;9:438. doi: 10.1038/s41597-022-01543-8 (PMC9308035; doi:10.1038/s41597-022-01543-8)
Supplement: Supplementary file 1 [file 41597_2022_1543_MOESM1_ESM.pdf]

Supplementary File 1. Examples illustrating the structure and coding scheme of the SARS-ANI Dataset.

Below are three examples illustrating the structure and coding scheme of the SARS-ANI Dataset. These examples intend to facilitate the comprehension of the data as well as the coding of potential relationships between events.

Table of Contents

Example 1 2

Example 2 3

Example 3 4

Example 1

The two following SARS-CoV-2 animal events occurred in the United States (**country_name**), in one household (**living_conditions =** *pet* AND event 2 is related to event 1, see **related_to_other_entries** = "living together") where three dogs were living (**number_susceptible =** *3*). The first dog was tested by PCR (**test =** *PCR*) because it had contact with a human diagnosed with COVID-19 (**reason_for_testing =** *confirmed human case*); the infection was confirmed on 2020-06-25 (**date_confirmed**): the dog showed symptoms that were not related to SARS-CoV-2 infection (**symptoms =** *unrelated symptoms*) and died from a cause not related to its SARS-CoV-2 status (**outcome** = *death not related to Sars-CoV-2*). This case was reported (**date_reported**) on 2020-07-02 by the WAHIS.

Almost two months later (**date_reported** = *2020-08-27*), a second dog of this household (counting at that time two dogs, **number_susceptible** = *2*) which had contact with the dog described in event 1 (**related_to_other_entries** = *living together* AND **reason_for_testing =** *confirmed animal case*) was tested for SARS-CoV-2 by virus neutralization test (**test**). The dog was asymptomatic (**symptoms =** *subclinica*l) and was isolated (**control_measures**). Outcome of the infection was not reported (outcome = *NS*).

Summary: These events describe one outbreak of SARS-CoV-2 in a multi-dog household following contact with a COVID-19-infected person and in which 2/3 dogs were infected (for clarity purpose, not all fields of the dataset are shown below).

ID	host_com_res	epidemiological_unit	number_cases	number_susceptible	number_tested	number_deaths
event 1	dog	animal	1	3	2	0
event 2	dog	animal	1	2	1	0

country_name	date_confirmed	date_reported	date_published	related_to_other_entries	related_ID	test
United States	2020-06-25	2020-07-02	2020-07-03	new	NA	PCR
United States	NS	2020-08-27	2020-08-27	living together	event 1	virus neutralisation test

sampling_type	reason_for_testing	symptoms	outcome	living_conditions	source_of_infection	control_measures
NS	confirmed human case	unrelated symptoms	death not related to Sars-CoV-2	pet	human	NA
NS	confirmed animal case	subclinical	NS	pet	animal	isolation

Example 2

The three following events occurred in a zoo (**living_conditions)** in the United States (**country_name**). A tiger living with four other animals from the same species (**number_susceptible** = 1+4 = *5*) was confirmed positive with SARS-CoV-2 by PCR (**test** = *PCR*) on 2020-04-03. Event 1 described infection in one animal (**epidemiological_unit** = *animal*). Date of reporting (**date_reported**) and publishing (**date_published**) are similar (*2020-04-06*). Condition of this tiger improved (**outcome** = *improved condition*).

Later in the month, two other events were reported in which SARS-CoV-2 infection was diagnosed by PCR (**test** = *PCR*) in the four other animals living with the tiger reported in event 3 (**related_to_other_entries** = *living together*). For both events, **date_confirmed** is not specified (*NS*), **date_reported** = *2020-04-17* and **date_published** = *2022-05-25*. For one animal (event 4), no information is available regarding the outcome of the infection (**outcome** = *NS*). For the three other animals described in event 5, the outcome of the infection is reported (**outcome** = *improved condition*). Because these three tigers were reported together and showed the same event and patients attributes (e.g. **date_reported**, **test**, **outcome**), they are entered as one event in the dataset (**epidemiological_unit** = *group*).

Summary: These three events describe one outbreak (occurrence of one or more cases in an epidemiological unit, which is here the enclosure of the tigers in the zoo), which started on 2020-04-03 and included five cases of SARS-CoV-2 infection (for clarity purpose, not all fields of the dataset are shown below).

ID	host_com_res	epidemiological_unit	number_cases	number_susceptible	number_tested
**event 3**	tiger	animal	1	5	1
**event 4**	tiger	animal	1	5	NS
**event 5**	tiger	group	3	5	NS

number_deaths	country_name	date_confirmed	date_reported	date_published	related_to_other_entries
0	United States	2020-04-03	2020-04-06	2020-04-06	new
0	United States	NS	2020-04-17	2020-04-25	living together
0	United States	NS	2020-04-17	2020-04-25	living together

related_ID	test	outcome	living_conditions	source_of_infection	variant
NA	PCR	improved condition	zoo	NS	NA
event 3	PCR	NS	zoo	NS	NA
event 3	PCR	improved condition	zoo	NS	NA

Example 3

The three following events report an outbreak that occurred in free ranging (**living_conditions** = *wildlife*) white-tailed deer (**host_com_res**) in Canada (**country**) that were tested during a surveillance/monitoring programme (**reason_for_testing**). The animals were sampled as part of the same epidemiological investigation but not necessary at the same geographic location (**epidemiological_unit** = *survey group*). Event 7 is an *update of* (**related_to_other_entries**) event 6 (**related_to_other_entries** = *update by*) and event 8 (related to event 7, see below) should also be considered as an *update of* event 6.

Events 6 and 7 report infections in more than one animal (**epidemiological_unit** = *survey group*) while event 8 reports infection in one single animal (**epidemiological_unit** = *animal*) related to the group described in event 7 (**related_to_other_entries** = *same study*). Event 7 and 8 are distinguished because the tests conducted in the animal in event 8 differed from those performed on the four animals described in event 7.

Date at which infection was confirmed (**date_confirmed)** is not specified in any events (*NS*); only event 6 was reported by the WAHIS (**date_reported** = *2022-01-20*). The follow-up events (events 7 and 8) were published ~1.5 month after event 6. Most likely, this difference is due to the time required for laboratory analyses (events 7 and 8 present more sample and test types and provide identification of the SARS-CoV-2 variant involved).

Summary: These three events describe one outbreak (occurrence of one or more cases in an epidemiological unit, which is here the surveyed animals) which was reported for the first time by WAHIS and published on 2022-01-20 and was subsequently updated on 2022-03-03 in ProMED-mail, probably following more laboratory investigations. The outbreak included five confirmed cases of SARS-CoV-2 in white-tailed deer in Canada. The five animals did not die following SARS-CoV-2 infection (**number_deaths** = *0* AND **outcome =** *death not related to Sars-CoV-2*). Indeed, they died from hunting, which this is not specified in the dataset but can be found in the reports (for clarity purpose, not all fields of the dataset are shown below).

ID	host_com_res	epidemiological_unit	number_cases	number_tested	number_deaths
event 6	white-tailed deer	survey group	5	213	0
event 7	white-tailed deer	survey group	4	213	0
event 8	white-tailed deer	animal	1	213	0

country_name	date_confirmed	date_reported	date_published	related_to_other_entries	related_ID
Canada	NS	2022-01-20	2022-01-20	updated by	event 7
Canada	NS	NA	2022-03-03	update of	event 6
Canada	NS	NA	2022-03-03	same study	event 7

test	sampling_type	test_2	sampling_type_2	test_3	sampling_type_3
PCR	NS	NA	NA	NA	NA
PCR	lymph node (post mortem)	PCR	nasal swab	gene sequencing	nasal swab
PCR	nasal swab	gene sequencing	nasal swab	NA	NA

reason_for_testing	symptoms	outcome	living_conditions	variant
surveillance/monitoring	subclinical	death not related to Sars-CoV-2	wildlife	NA
surveillance/monitoring	subclinical	death not related to Sars-CoV-2	wildlife	Ontario WTD lineage
surveillance/monitoring	subclinical	death not related to Sars-CoV-2	wildlife	Ontario WTD lineage
